# Texture Analysis Using CT and Chemical Shift Encoding-Based Water-Fat MRI Can Improve Differentiation Between Patients With and Without Osteoporotic Vertebral Fractures

**DOI:** 10.3389/fendo.2021.778537

**Published:** 2022-01-04

**Authors:** Nico Sollmann, Edoardo A. Becherucci, Christof Boehm, Malek El Husseini, Stefan Ruschke, Egon Burian, Jan S. Kirschke, Thomas M. Link, Karupppasamy Subburaj, Dimitrios C. Karampinos, Roland Krug, Thomas Baum, Michael Dieckmeyer

**Affiliations:** ^1^ Department of Diagnostic and Interventional Radiology, University Hospital Ulm, Ulm, Germany; ^2^ Department of Radiology and Biomedical Imaging, University of California San Francisco, San Francisco, CA, United States; ^3^ Department of Diagnostic and Interventional Neuroradiology, School of Medicine, Klinikum rechts der Isar, Technical University of Munich, Munich, Germany; ^4^ TUM-Neuroimaging Center, Klinikum rechts der Isar, Technical University of Munich, Munich, Germany; ^5^ Department of Diagnostic and Interventional Radiology, School of Medicine, Klinikum rechts der Isar, Technical University of Munich, Munich, Germany; ^6^ Engineering Product Development (EPD) Pillar, Singapore University of Technology and Design (SUTD), Singapore, Singapore; ^7^ Changi General Hospital, Singapore, Singapore

**Keywords:** bone mineral density, convolutional neural network, opportunistic imaging, osteoporosis, proton density fat fraction, texture analysis, vertebral fracture

## Abstract

**Purpose:**

Osteoporosis is a highly prevalent skeletal disease that frequently entails vertebral fractures. Areal bone mineral density (BMD) derived from dual-energy X-ray absorptiometry (DXA) is the reference standard, but has well-known limitations. Texture analysis can provide surrogate markers of tissue microstructure based on computed tomography (CT) or magnetic resonance imaging (MRI) data of the spine, thus potentially improving fracture risk estimation beyond areal BMD. However, it is largely unknown whether MRI-derived texture analysis can predict volumetric BMD (vBMD), or whether a model incorporating texture analysis based on CT and MRI may be capable of differentiating between patients with and without osteoporotic vertebral fractures.

**Materials and Methods:**

Twenty-six patients (15 females, median age: 73 years, 11 patients showing at least one osteoporotic vertebral fracture) who had CT and 3-Tesla chemical shift encoding-based water-fat MRI (CSE-MRI) available were analyzed. In total, 171 vertebral bodies of the thoracolumbar spine were segmented using an automatic convolutional neural network (CNN)-based framework, followed by extraction of integral and trabecular vBMD using CT data. For CSE-MRI, manual segmentation of vertebral bodies and consecutive extraction of the mean proton density fat fraction (PDFF) and T2* was performed. First-order, second-order, and higher-order texture features were derived from texture analysis using CT and CSE-MRI data. Stepwise multivariate linear regression models were computed using integral vBMD and fracture status as dependent variables.

**Results:**

Patients with osteoporotic vertebral fractures showed significantly lower integral and trabecular vBMD when compared to patients without fractures (p<0.001). For the model with integral vBMD as the dependent variable, T2* combined with three PDFF-based texture features explained 40% of the variance (adjusted R^2^ $\left[R_{a}^{2}\right]$ = 0.40; p<0.001). Furthermore, regarding the differentiation between patients with and without osteoporotic vertebral fractures, a model including texture features from CT and CSE-MRI data showed better performance than a model based on integral vBMD and PDFF only ( $R_{a}^{2}$ = 0.47 vs. $R_{a}^{2}$ = 0.81; included texture features in the final model: integral vBMD, CT_Short-run_emphasis, CT_Varianceglobal, and PDFF_Variance).

**Conclusion:**

Using texture analysis for spine CT and CSE-MRI can facilitate the differentiation between patients with and without osteoporotic vertebral fractures, implicating that future fracture prediction in osteoporosis may be improved.

## Introduction

In clinical routine, assessment of areal bone mineral density (BMD) and the individual risk of bone fracture is commonly obtained using dual-energy X-ray absorptiometry (DXA) (1–4). Fractures due to low bone mass and microarchitectural deterioration are characteristic for osteoporosis, a systemic skeletal disease with very high prevalence worldwide (1, 4–7). Osteoporotic fragility fractures can severely impair the health-related quality of life and have been linked to premature mortality (1, 8–10). Among osteoporotic fragility fractures, vertebral fractures are particularly prevalent, with an estimated 12.6-fold increase in the risk of future additional vertebral fractures (1, 11–13). A major clinical issue of these fractures is that they occur frequently but can remain asymptomatic for a long time (13, 14). This can delay diagnosis, timely treatment initiation, and subsequent approaches to avoid future additional osteoporotic vertebral fractures (13, 14).

Clinical management requires a reliable assessment of the individual fracture risk in each patient, but DXA is known to have inherent limitations including inaccuracy in differentiating patients with and without prevalent vertebral fractures, in predicting new vertebral fractures, and for treatment monitoring in osteoporosis (15–17). Hence, imaging alternatives to DXA are needed to improve patient care, which include computed tomography (CT) and magnetic resonance imaging (MRI) (18–21). Using CT opportunistically for the assessment of volumetric BMD (vBMD) has become increasingly popular (i.e., extraction of the vBMD from routine CT data acquired for other purposes than osteoporosis screening, such as staging in oncologic patients), using conversion equations for translation of Hounsfield units (HU) into vBMD, showing overall good correlations with DXA measurements and sufficient reproducibility (18, 22–25). Regarding MRI, particularly chemical shift encoding-based water-fat MRI (CSE-MRI) has developed into a valuable tool to determine a vertebral body’s proton density fat fraction (PDFF), which is considered a surrogate biomarker of bone health (19, 21). As such, the PDFF from CSE-MRI represents a quantitative, accurate, and robust marker of a tissue’s relative fat content (26–30). The PDFF is defined as the ratio of density of mobile protons from fat (triglycerides) and the total density of protons from mobile triglycerides and mobile water and is a fundamental property of tissue (29). Osteoporosis has been linked to increased fat content of bone marrow, given that with aging, the composition of bone marrow shifts to favor the presence of adipocytes, while osteoclast activity increases and osteoblast function declines, leading to osteoporosis (31). Thus, increased PDFF can be observed in relation to osteoporosis, and inverse correlations with DXA- and CT-derived BMD have been reported (19, 32–34). However, a patient’s susceptibility to fragility fractures is not solely explained by decreased BMD or alterations in fat content of bone marrow. Importantly, bone strength and resistance to fracture are also determined by other factors, including bone geometry and microstructural architecture (35).

Texture analysis has been applied as an advanced image analysis technique to provide a spatially resolved evaluation of osseous structures like the vertebral body in osteoporosis (18, 19, 36). In essence, texture analysis provides spatial and heterogeneity information of gray-level values in images, representing an objective and quantitative approach to analyze the distribution and relationship of pixel or voxel gray levels (37–39). In spine imaging, commonly used texture features include first-order, second-order, and higher-order features, and their extraction has been achieved for sagittal reformations with up to 3 mm slice thickness for routine multi-detector CT, and, more recently, also for CSE-MRI (40, 41). Yet, it is largely unknown whether CSE-MRI-derived texture analysis can predict vBMD, or whether a model incorporating texture analysis based on both CT and CSE-MRI in the same patients may lead to improved capability for differentiating patients with and without osteoporotic vertebral fractures.

Against this background, the main purposes of this study were to apply texture analysis to CSE-MRI data of the thoracolumbar spine to predict vBMD, and to facilitate discrimination between patients with and without osteoporotic vertebral fractures by implementing texture analysis. We hypothesize that combining CT- and CSE-MRI-based texture analysis may result in improved differentiation between patients with and without osteoporotic vertebral fractures compared to vBMD and PDFF alone.

## Material and Methods

### Study Inclusion and Patient Cohort

This retrospective study was approved by the local institutional review board and was conducted in accordance with the Declaration of Helsinki. The requirement for written informed consent was waived due to the study’s retrospective character.

Eligible patients underwent CT and MRI scans covering the thoracolumbar spine and were identified in our institution’s digital Picture Archiving and Communication System (PACS). Inclusion criteria were as follows: 1) age of at least 18 years, 2) acquisition of routine CT and CSE-MRI within a maximum of six months, and 3) overlap in spatial coverage between CT and CSE-MRI of at least two consecutive vertebral bodies. The exclusion criteria were as follows: 1) malignant bone lesions (e.g., vertebral bone metastases), 2) hematological or metabolic bone disorders aside from osteoporosis, and 3) motion artifacts in imaging data. Patients who had undergone previous surgery at the spine and showed spinal instrumentation were included, but vertebral bodies with foreign material were excluded from the analyses (due to metal-related artifacts in imaging data). Both CT and CSE-MRI were acquired for standard routine clinical indications (e.g., lumbar back pain, screening for vertebral fractures).

**Figure 1:**
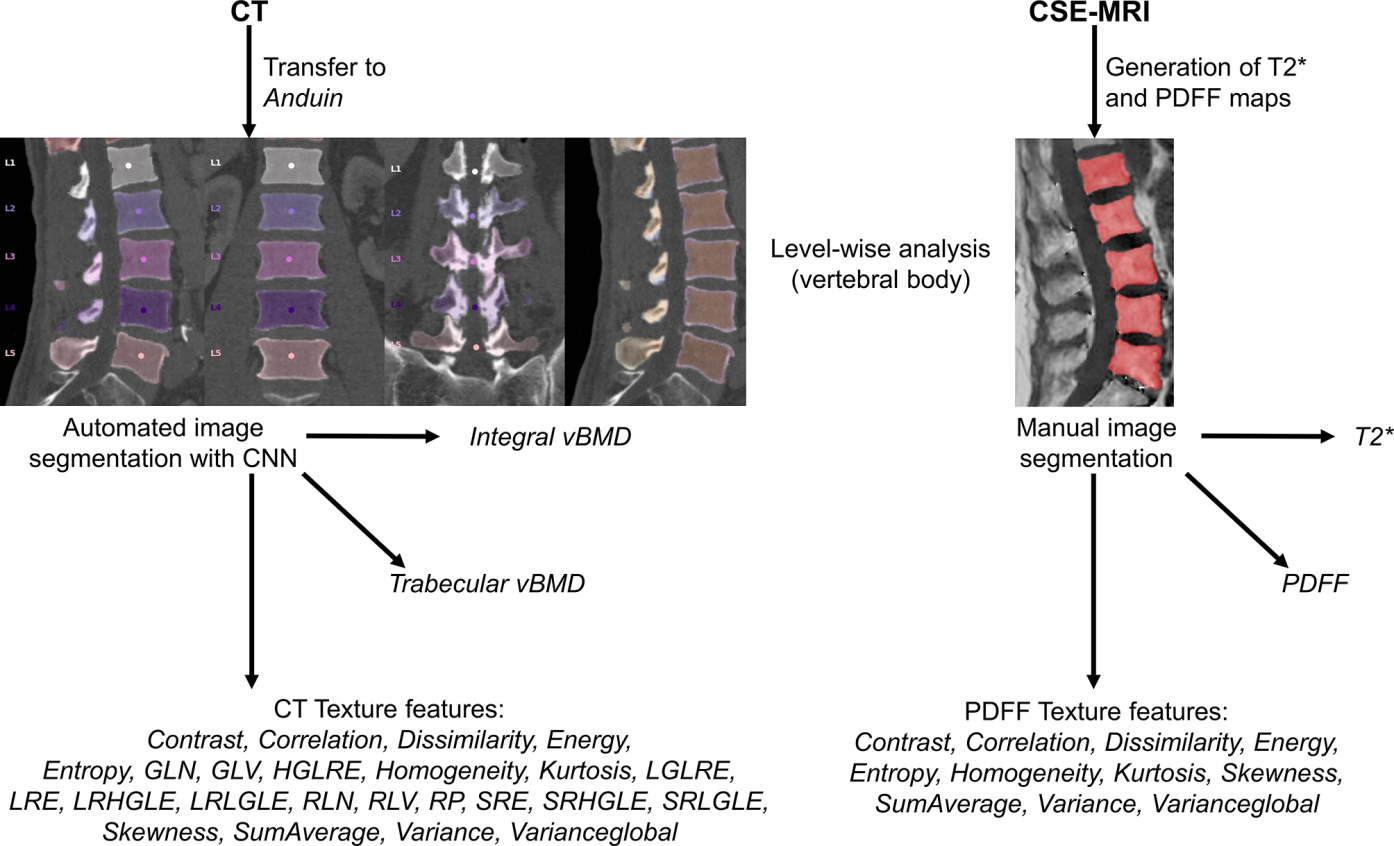
Overview of the study setup. In this study, texture analysis to extract different texture features was achieved based on computed tomography (CT) and chemical shift encoding-based water-fat magnetic resonance imaging (CSE-MRI) data. For labeling and segmentation of vertebral bodies from CT data, a convolutional neural network (CNN)-based framework was used (https://anduin.bonescreen.de). After generation of proton density fat fraction (PDFF) and T2* maps, vertebral bodies were segmented manually on the PDFF maps. In addition to different texture features, integral and trabecular volumetric bone mineral density (vBMD) were extracted from segmented CT data, and mean PDFF and T2* values were extracted level-wise from segmented CSE-MRI data.

Overall, 26 patients fulfilled the eligibility criteria and underwent imaging at the same institution between July 2018 and September 2019. **Figure 1** provides an overview of the data processing pipeline.

### Computed Tomography

#### Image Acquisition

Scanning was performed with five different CT scanners (Philips iQon, iCT 256, Ingenuity, Ingenuity Core, Philips Healthcare, Best, The Netherlands; Somatom Definition AS+, Siemens Healthineers, Erlangen, Germany). Scans in five of the included patients were performed after administration of either both oral (Barilux Scan, Sanochemia Diagnostics) and intravenous (Iomeron 400, Bracco) contrast media, or only after administration of an intravenous contrast medium. The contrast-enhanced scans were acquired either in the arterial or portal venous phase, triggered by a threshold of CT attenuation surpassed in a region of interest (ROI) placed in the aorta or after a delay of 70 s, respectively, which was dependent on the distinct clinical indication for imaging.

Image data were acquired with all scanners in helical mode (using asynchronous calibration), a slice thickness of 0.9 to 1 mm, and adaptive tube load. The median tube voltage was 120 kV (range: 120 – 140 kV), the median tube current amounted to 316 mA (range: 118 – 363 mA). Sagittal reformations of the spine using a bone kernel were either available with a slice thickness of 2 mm (18 patients) or 3 mm (8 patients). The presence of vertebral fractures was determined in sagittal reformations of the spine with a bone kernel by a board-certified radiologist with eleven years of experience (T.B.), using the classification system described by Genant et al. (42).

#### Image Processing and Segmentation

**Figure 2:**
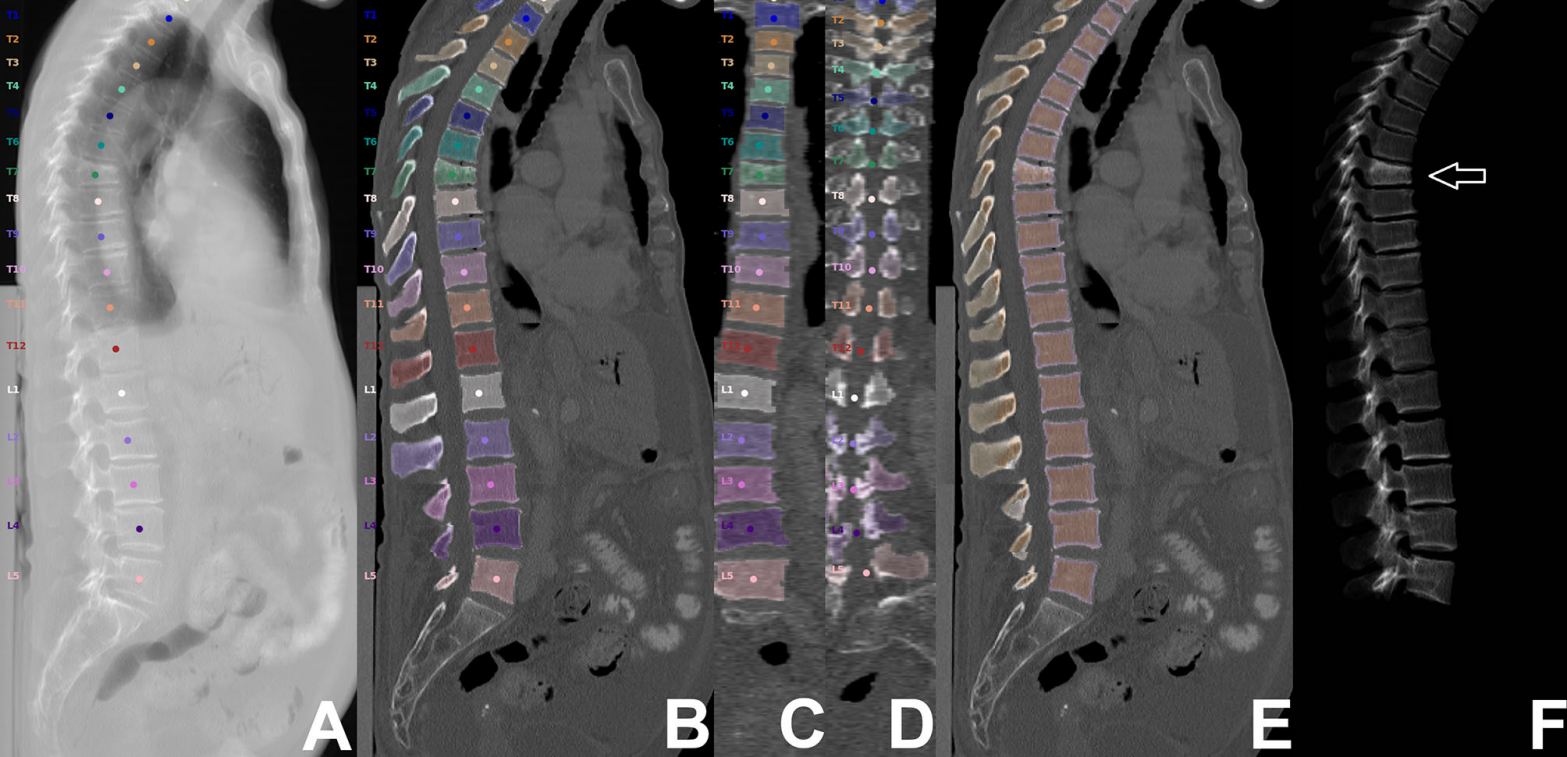
Segmentation of computed tomography (CT) data. CT scan of a 63-year-old woman visualized as virtual radiograph-like images in lateral projection **(A, F)** and as planar reconstructions in lateral and coronal views **(B–E)**, covering the thoracolumbar spine (T1-L5). Labeling and segmentation of single vertebrae and vertebral subregions was achieved automatically using a convolutional neural network (CNN)-based framework. Segmentation masks were used to extract integral volumetric bone mineral density (vBMD), trabecular vBMD, and texture features following texture analysis. However, this patient showed a vertebral fracture at level T7 (white arrow); thus, extraction of these parameters was not performed for this vertebral body.

From PACS, images were transferred to our in-house developed, convolutional neural network (CNN)-based framework (https://anduin.bonescreen.de) (**Figures 1** and **2**) (43–45). This tool identifies and labels each vertebra in an automated process, followed by creating corresponding segmentation masks for each vertebra as well as its subregions. Furthermore, it adjusts for the used scanner, scanning parameters, and contrast media (if administered during imaging) to asynchronously convert measured HU into vBMD (43–45).

For this study, we used segmentation masks of the trabecular and cortical compartment of the vertebral body, respectively. By combining the trabecular and cortical compartment, the entire vertebral body was enclosed. The generated labels and segmentation masks of all vertebrae were checked visually by a radiologist (two years of experience in spine imaging, M.D.), and were manually corrected if necessary (e.g., in case of labeling or segmentation errors due to Schmorl nodes, fused vertebral bodies, or thoracolumbar or lumbosacral transitional anatomy). Then, the segmentation masks of the entire vertebral bodies were used for level-wise extraction of integral vBMD, and subregion masks of the trabecular compartment were used for sampling of trabecular vBMD. All thoracolumbar vertebral bodies without foreign material (e.g., due to spinal instrumentation), severe degenerative changes (e.g, Modic type 3 endplate changes), or vertebral fractures were considered for integral and trabecular vBMD extractions.

### Magnetic Resonance Imaging

#### Image Acquisition

All patients were scanned using the same 3-Tesla MRI system (Ingenia, Philips Healthcare, Best, The Netherlands). The pulse sequence protocol was individually determined based on the clinical indication for imaging, but included a sagittal six-echo time (TE)-interleaved three-dimensional (3D) spoiled gradient echo sequence for CSE-MRI (46).

Imaging was performed in supine position using the built-in-the-table posterior coil elements (12-channel array). The six echoes of the CSE-MRI sequence were acquired in two interleaves acquiring 3 echoes per repetition time (TR), using flyback (monopolar) read-out gradients and the following imaging parameters: TR/TE1/effective ΔTE = 8.3/1.32/1.0 ms, acquisition matrix size = 124 × 122 x 69, acquisition voxel size = 1.80 mm × 1.80 mm × 1.80 mm, receiver bandwidth = 1,083 Hz/pixel, frequency direction = anterior-posterior, 1 average, approximate scan time = 3 min 38 s. A flip angle of 3° was used to minimize T1-bias effects (47, 48).

#### Image Processing and Segmentation

Reconstruction of raw data, fat quantification, and T2* mapping were performed offline. The MATLAB-based Reconstruction Library (MRecon/ReconFrame, version 4.3.3; https://www.gyrotools.com/gt/index.php/products/reconframe with MATLAB, version R2021a; MathWorks Inc., Natick, MA, USA) was used for raw data reconstruction. Water-fat separation was performed using a graph-cut algorithm, employing a multi-peak fat model specific to bone marrow and a single T2* decay model (49, 50). The PDFF maps were computed as the ratio of the fat signal over the sum of fat and water signals (29, 51, 52). T2* maps were extracted in addition to PDFF maps.

**Figure 3:**
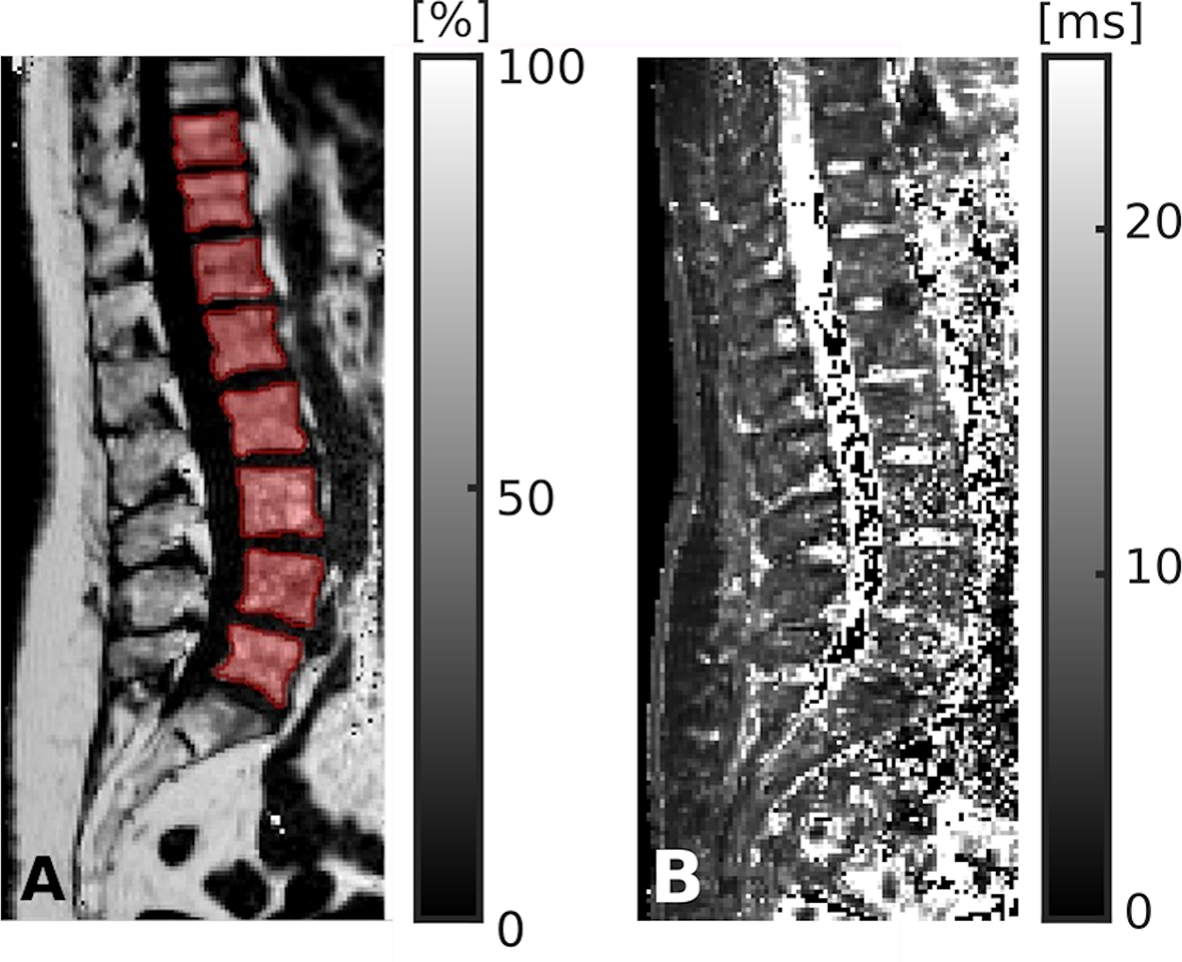
Segmentation of chemical shift encoding-based water-fat magnetic resonance imaging (CSE-MRI) data. CSE-MRI scan of a 74-year-old woman: proton density fat fraction (PDFF) map [%] with manually prescribed segmentation masks **(A)** and T2* map [ms] **(B)** covering the lower thoracic and lumbar vertebral bodies (T10-L5). Segmentation masks were used to extract PDFF, T2*, and texture features following texture analysis.

The vertebral bodies were manually segmented using sagittal images. In the reconstructed maps, manual polygonal ROIs were carefully placed to enclose the vertebral body using MITK ([http://mitk.org/wiki/The_Medical_Imaging_Interaction_Toolkit_(MITK)]; German Cancer Research Center, Division of Medical and Biological Informatics, Medical Imaging Interaction Toolkit, Heidelberg, Germany; **Figures 1** and **3**) (51, 52). The segmentations did not include the posterior elements and were performed by the same radiologist who had also performed visual inspection of the results of the CNN-based algorithm for CT data processing (two years of experience in spine imaging, M.D.). Level-wise PDFF and T2* values were then extracted from the segmentation masks for those vertebral bodies that were also depicted and considered during CT data analyses.

### Texture Analysis

On the basis of the distribution of gray-level values, texture analysis was used to characterize structural image properties of predefined regions by quantifying different texture features (37–39). Texture analysis was performed using the segmentation masks derived from CT and CSE-MRI enclosing single vertebral bodies, and different first-order statistical moments from global gray-level histograms, second-order features based on the gray-level co-occurrence matrix (GLCM), and higher-order features based on the gray-level run-length matrix (GLRLM) were extracted (**Figure 1** and **Table 1**) (40, 41, 51, 53–55).

**Table 1:** Texture features derived from computed tomography (CT) and chemical shift encoding-based water-fat magnetic resonance imaging (CSE-MRI).

Category	Texture Feature	Description	Included in CT-Based Texture Analysis	Included in CSE-MRI-Based Texture Analysis
Global	Varianceglobal	Spread of gray-level distribution	x	x
	Skewness	Shape of gray-level distribution	x	x
	Kurtosis	Flatness of gray-level distribution	x	x
Second-order (GLCM)	Energy	Uniformity	x	x
	Contrast	Local intensity variation	x	x
	Entropy	Randomness	x	x
	Homogeneity	Homogeneous scene	x	x
	Correlation	Linear spatial relationships between texture elements	x	x
	SumAverage	Spread of the mean voxel co-occurrence distribution	x	x
	Variance	Voxel co-occurrence distribution	x	x
	Dissimilarity	Heterogeneity	x	x
Higher-order (GLRLM)	SRE	Short-run emphasis	x	
	LRE	Long-run distribution	x	
	GLN	Similarities of gray-levels	x	
	RLN	Similarity in length of runs	x	
	RP	Distribution and homogeneity of runs with a specific direction	x	
	LGLRE	Distribution of low gray-level values	x	
	HGLRE	Distribution of high gray-level values	x	
	SRLGLE	Joint distribution of short runs and low gray-level values	x	
	SRHGLE	Joint distribution of short runs and high gray-level values	x	
	LRLGLE	Joint distribution of long runs and low gray-level values	x	
	LRHGLE	Joint distribution of long runs and high gray-level values	x	
	GLV	Weighted variances of gray-level values	x	
	RLV	Weighted variances of gray-level runs	x	

Global (histogram-based), gray-level co-occurrence matrix (GLCM)-based, and gray-level run-length matrix (GLRLM)-based texture features and their descriptions. The table provides information about which texture features were considered for texture analysis based on computed tomography (CT) and chemical shift encoding-based water-fat magnetic resonance imaging (CSE-MRI) data.

SRE, short-run emphasis; LRE, long-run emphasis; GLN, gray-level non-uniformity; RLN, run-length non-uniformity; RP, run percentage; LGLRE, low gray-level run emphasis; HGLRE, high gray-level run emphasis; SRLGLE, short-run low gray-level emphasis; SRHGLE, short-run high gray-level emphasis; LRLGLE, long-run low gray-level emphasis; LRHGLE, long-run high gray-level emphasis; GLV, gray-level variance; RLV, run-length variance.

The GLCM reflects how often pairs of voxels with a given gray-level value and offset occur in an image. The entries of the GLCMs at different angular directions θ = (0°, 45°, 90°, and 135°) were generated by computing the joint probability of two adjacent voxel intensities at a given offset d = (dx, dy, dz) and given θ, with dx, dy, and dz denoting the displacement along the x-, y-, and z-axis, respectively. 3D GLCM analysis was achieved by computation of the co-occurrence probabilities of voxel intensities from the 26 neighbors aligned in 13 directions. Averaging over the 13 directions ensures rotation invariance. Furthermore, a gray-level run is defined as a set of successive voxels with identical gray-level values that are arranged collinearly in a certain direction, and the run-length represents the number of voxels in it. The GLRLM features are calculated based on the occurrence and distribution of such runs within the GLCM and measure directional changes in the GLCM. Analogously, 3D GLRLM was computed by simultaneously adding up all possible run-lengths in the 13 directions of the 3D space. For GLCM and GLRLM analysis, direction-dependent discretization length differences were taken into account when measurements were combined by averaging or summation. All steps of the texture analysis were performed with MATLAB (version R2021a; MathWorks Inc., Natick, MA, USA) using a radiomics toolbox (https://github.com/mvallieres/radiomics).

### Statistical Analysis

For statistical analyses, SPSS (version 26.0; IBM SPSS Statistics for Windows, IBM Corp., Armonk, NY, USA) and GraphPad Prism (version 6.0; GraphPad Software Inc., San Diego, CA, USA) were used. A p-value of < 0.05 (two-sided) was considered statistically significant.

Descriptive statistics were calculated for integral and trabecular vBMD, PDFF, T2*, and the different texture features extracted from CT and CSE-MRI data, respectively. Shapiro-Wilk normality tests indicated non-Gaussian distribution for the majority of these parameters. Integral and trabecular vBMD were compared between patients with and without osteoporotic vertebral fractures using Mann-Whitney tests. Furthermore, age and sex distributions were compared between the subgroups of patients with and without osteoporotic vertebral fractures using Mann-Whitney and Chi-squared tests, respectively.

For vertebral level-wise analyses, data from each vertebral body were considered as a separate data point. One stepwise linear regression model using integral vBMD as the dependent and PDFF, T2*, and all CSE-MRI-derived texture features as independent variables was calculated. Furthermore, two additional linear regression models with a stepwise approach were generated, using the binary fracture status (at least one osteoporotic vertebral fracture present/no osteoporotic vertebral fracture present) as the dependent and 1) integral vBMD, PDFF, and T2*, or 2) integral vBMD, PDFF, T2*, and all CT-derived as well as the CSE-MRI-derived texture features as independent variables. Patient age, sex, the number of independent variables, and the vertebral level (T1-L5) were considered for adjustment of these regression models.

Furthermore, integral and trabecular vBMD, PDFF, T2*, and texture features derived from CT and CSE-MRI were averaged over the included vertebral bodies to provide one value per parameter in each patient, respectively. Using these mean values, two additional linear regression models with a stepwise approach were computed, using again the binary fracture status (at least one osteoporotic vertebral fracture present/no osteoporotic vertebral fracture present) as the dependent and 1) integral vBMD, PDFF, and T2*, or 2) integral vBMD, PDFF, T2*, and all CT-derived as well as the CSE-MRI-derived texture features as independent variables. Patient age, sex, and the number of independent variables were used for adjustment of these regression models.

Independent variables were included stepwise in the linear regression models based on a p-value threshold of < 0.05. Per model, the adjusted R^2^ $\left(R_{a}^{2}\right)$ is reported with related β coefficients and 95%-confidence intervals (CIs), and F-tests were performed to assess statistical significance of the final models after stepwise inclusion of variables.

## Results

### Patient Characteristics

Analyses included 171 vertebral bodies of the thoracolumbar spine, which were derived from 26 patients (15 females, 11 males, median age: 73 years, age range: 29 – 86 years). Eight of the included patients had imaging by CT and CSE-MRI on the same day, the median interval between CT and CSE-MRI acquisitions was four days.

Eleven of the included patients (42.3%) showed at least one osteoporotic vertebral fracture according to CT image reading (only Genant grade 1 fractures: 2 patients, only Genant grade 2 fractures: 2 patients, combination of fracture grades including Genant grade 3 fractures: 7 patients). Patients with and without vertebral fractures did not statistically significantly differ in age (patients with fractures: median age: 75.1 years, age range: 59 – 86 years, patients without fractures: median age: 71.5 years, age range: 29 – 78 years; p = 0.097) or sex distributions (patients with fractures: 3 males & 8 females, patients without fractures: 8 males & 7 females; p = 0.184).

Regarding the analyzed thoracic vertebrae (89 thoracic vertebral bodies in total), T1 was considered in five patients, T2, T3, and T4 in six patients, T5, T6, T10, and T11 in seven patients, T7, T8, and T9 in eight patients, and T12 in 14 patients, respectively. Furthermore, for the lumbar spine (82 lumbar vertebral bodies in total), L1 and L4 were included from 18 patients each, while L2 was considered in 17 patients, L3 in 14 patients, and L5 in 15 patients, respectively.

### Volumetric Bone Mineral Density

Patients with osteoporotic vertebral fractures had statistically significantly lower integral and trabecular vBMD as compared to patients without osteoporotic vertebral fractures regarding all included vertebrae (integral vBMD: 153.1 ± 46.5 mg/cm^3^ vs. 218.5 ± 45.4 mg/cm^3^; p < 0.001; trabecular vBMD: 77.7 ± 30.3 mg/cm^3^ vs. 130.8 ± 34.8 mg/cm^3^; p < 0.001). The minimum value measured for a vertebral body in patients with an osteoporotic vertebral fracture was 82.6 mg/cm^3^ for integral vBMD and 21.6 mg/cm^3^ for trabecular vBMD, compared to 132.4 mg/cm^3^ for integral vBMD and 71.8 mg/cm^3^ for trabecular vBMD in patients without osteoporotic vertebral fractures.

### Prediction of Volumetric Bone Mineral Density and Differentiation Between Patients According to Fracture Status

For the model with integral vBMD as the dependent variable (independent variables: PDFF, T2*, and texture features derived from CSE-MRI), $R_{a}^{2}$ amounted to 0.40 (F(7, 163) = 17.5, p < 0.001), with the following variables being kept in the final model after the stepwise approach: T2*, PDFF_Kurtosis (global texture feature, representing the flatness of gray-level distribution), PDFF_Variance (second-order texture feature, representing the voxel co-occurrence distribution), and PDFF_Energy (second-order texture feature, representing uniformity).

For vertebral level-wise analyses with the fracture status as the dependent variable (independent variables: integral vBMD, PDFF, and T2*), $R_{a}^{2}$ of 0.44 was obtained (F(5, 165) = 27.4, p < 0.001; variables included in the final model: vBMD and PDFF). Including the texture features in the model (independent variables: integral vBMD, PDFF, T2*, texture features derived from CT and CSE-MRI) resulted in a statistically significant model with an improved $R_{a}^{2}$ of 0.66 (F(10, 160) = 34.7, p < 0.001; **Table 2**). Specifically, the variables included in the final model were CT_Correlation (second-order texture feature, representing the linear spatial relationships between texture elements), CT_SRLGLE (higher-order texture feature, representing the joint distribution of short runs and low gray-level values), PDFF_SumAverage (second-order texture feature, representing the spread of the mean voxel co-occurrence distribution), CT_Varianceglobal (global texture feature, representing the spread of gray-level distribution), CT_LRHGLE (higher-order texture feature, representing the joint distribution of long runs and high gray-level values), CT_Contrast (second-order texture feature, representing the local intensity variation), and PDFF_Energy (second-order texture feature, representing uniformity).

**Table 2:** Differentiation between patients with and without osteoporotic vertebral fractures including texture analysis – analysis on vertebral level.

Term	Description	β coefficient	95%-CI	p-value
CT_Correlation	Second-order texture feature, representing the linear spatial relationships between texture elements	-0.639	-1.308;-0.938	<0.001
CT_SRLGLE	Higher-order texture feature, representing the joint distribution of short runs and low gray-level values	0.173	-44.109;2028.023	0.060
PDFF_SumAverage	Second-order texture feature, representing the spread of the mean voxel co-occurrence distribution	-0.183	-134.472;-30.952	0.002
CT_Varianceglobal	Global texture feature, representing the spread of gray-level distribution	-0.435	-0.020;-0.005	0.001
CT_LRHGLE	Higher-order texture feature, representing the joint distribution of long runs and high gray-level values	-0.724	0.000;0.000	<0.001
CT_Contrast	Second-order texture feature, representing the local intensity variation	0.551	0.000;0.000	<0.001
PDFF_Energy	Second-order texture feature, representing uniformity	-0.201	-227.524;-36.375	0.007

This table shows the variables kept in the final linear regression model (adjusted R^2^ [R^2^_a_] = 0.66, (F(10, 160) = 34.7, p < 0.001) after a stepwise approach using the binary fracture status (at least one osteoporotic vertebral fracture present/no osteoporotic vertebral fracture present) as the dependent variable (vertebral level-wise analyses). Specifically, it included the texture features CT_Correlation, CT_SRLGLE, PDFF_SumAverage, CT_Varianceglobal, CT_LRHGLE, CT_Contrast, and PDFF_Energy (β coefficients, 95%-confidence intervals [CIs], and p-values shown per texture feature). Patient age, sex, the number of independent variables, and the vertebral level (T1-L5) were considered for adjustment. For vertebral level-wise analyses, the data from each vertebral body were considered as a separate data point.

On a patient level, using the fracture status as the dependent variable (independent variables: integral vBMD, PDFF, and T2*) resulted in $R_{a}^{2}$ of 0.47 (F(4, 21) = 6.5, p = 0.001; variables included in final model: vBMD and PDFF). Comparable to the level-wise analyses, including texture analysis in the model (independent variables: integral vBMD, PDFF, T2*, texture features derived from CT and CSE-MRI) resulted in a statistically significant model with an improved $R_{a}^{2}$ of 0.81 (F(6, 19) = 19.2, p < 0.001, **Table 3)**. The variables included in the final model were integral vBMD, CT_SRE (higher-order texture feature, representing the short-run emphasis), CT_Varianceglobal (global texture feature, representing the spread of gray-level distribution), and PDFF_Variance (second-order texture feature, representing the voxel co-occurrence distribution).

**Table 3:** Differentiation between patients with and without osteoporotic vertebral fractures including texture analysis – analysis on patient level.

Term	Description	β coefficient	95%-CI	p-value
Integral vBMD	–	-0.669	-0.010;-0.005	<0.001
CT_SRE	Higher-order texture feature, representing the short-run emphasis	0.721	154.622;287.516	<0.001
CT_Varianceglobal	Global texture feature, representing the spread of gray-level distribution	-0.519	-0.021;-0.008	<0.001
PDFF_Variance	Second-order texture feature, representing the voxel co-occurrence distribution	0.351	5.390;36.408	0.011

This table shows the variables kept in the final linear regression model (adjusted R^2^ [R^2^_a_] = 0.81 (F(6, 19) = 19.2, p < 0.001) after a stepwise approach using the binary fracture status (at least one osteoporotic vertebral fracture present/no osteoporotic vertebral fracture present) as the dependent variable (analyses on patient level). Specifically, it included integral volumetric bone mineral density (vBMD) and the texture features CT_SRE, CT_Varianceglobal, and PDFF_Variance (β coefficients, 95%-confidence intervals [CIs], and p-values shown per texture feature). Patient age, sex, and the number of independent variables were considered for adjustment. For analyses on patient level, integral and trabecular vBMD, PDFF, T2*, and texture features were averaged over the included vertebral bodies to provide one value per parameter in each patient, respectively.

## Discussion

In this study, we used texture analysis on CT and CSE-MRI data covering the thoracolumbar spine in patients with and without osteoporotic vertebral fractures to predict vBMD, and to discriminate between patients with and without osteoporotic vertebral fractures based on models including vBMD, PDFF, T2*, and texture features. The main findings are as follow: first, a model including T2* combined with three PDFF-based texture features explained 40% of the variance in integral vBMD; second, a model consisting of integral vBMD and three texture features (CT_SRE, CT_Varianceglobal, and PDFF_Variance) explained 81% of the variance regarding the osteoporotic vertebral fracture status, compared to 47% when the model was based on integral vBMD and PDFF only.

Main complications of osteoporosis are vertebral fractures as a result of decreased bone strength, which is determined by a multitude of factors such as bone geometry, cortical thickness and porosity, trabecular bone morphology, and intrinsic properties of bony tissue (35). Of note, DXA as the reference standard for measuring the areal BMD and assessing individual fracture risk cannot fully capture many of those factors, thus harboring well-known limitations for predicting new osteoporotic vertebral fractures or differentiating between patients with and without prevalent vertebral fractures, as well as for treatment monitoring (15–17). In this regard, it is estimated that DXA-based areal BMD values only account for about 60 to 70% of the variation in bone strength (35). Yet, texture analysis based on CT or MRI data can provide spatially resolved assessments of vertebral body composition, thus supplementing conventional vBMD measurements with parameters potentially valuable for improving image-based osteoporosis diagnostics and fracture prediction. Specifically, it has been demonstrated that texture analysis with a support vector machine can be performed opportunistically on routine CT data of the spine, potentially enabling to discriminate between patients depending on their fracture status (considering the texture features Energy, Entropy, and Homogeneity) (40). The support vector machine classifier operated with linear, polynomial, and radial basis function kernels to discriminate between healthy subjects and patients with fractures, while the radial basis function kernel revealed the best performance (sensitivity of 93.33%, specificity of 79.33%, and accuracy of 83% among different kernel functions) (40). Another study demonstrated that an improved classification of patients with and without prevalent vertebral fractures is possible by combining texture features with regional vBMD [area under the curve (AUC) = 0.88)], with global vBMD showing inferior performance (AUC = 0.64) (56). Likewise, a set of texture features derived from routine CT exams in a machine-learning approach identified patients who would suffer from vertebral fractures with high accuracy (AUC = 0.97) (57).

Recently, the feasibility of texture analysis based on CSE-MRI has been demonstrated, which could principally provide insights into bone health beyond mean PDFF of vertebral bodies (41, 51). Leveraging CSE-MRI by additionally using texture analysis has revealed that vertebral bone marrow heterogeneity is dependent on sex and age, and is increased in postmenopausal women, which is a patient cohort at particular risk for osteoporosis and related vertebral fractures (41, 51). Results of the present study show that a model incorporating T2* and three texture features based on CSE-MRI predicted the variation in integral vBMD by a proportion of 40%, while PDFF alone was no significant predictor in the model. This may be regarded as evidence for the need for more advanced analyses of CSE-MRI data beyond mere PDFF. However, further improvement in explanatory power for vBMD variance based on CSE-MRI-derived parameters may not be feasible, given that primarily mineralized tissue (trabecular and cortical bone) contributes to the vBMD, while PDFF primarily captures the vertebral bone marrow located in the cavities of trabecular bone (18, 19, 31). Increased PDFF in osteoporosis and inverse correlations with DXA- or CT-derived BMD have been previously reported, though (19, 32–34). Moreover, T2* was kept in the model, which can be considered an MRI-based parameter related to bone microstructure and density (58–60). Specifically, it has been demonstrated that T2* correlates with the density and orientation of trabecular bone (59). Thus, explanation to a certain degree of the variance in vBMD by a model including T2* seems reasonable. In this regard, recent work presented a 3D adiabatic inversion recovery prepared ultra-short echo-time (UTE) Cones sequence for direct volumetric imaging of trabecular bone of the human spine that is robust in suppressing both water and fat, and can provide high image contrast for short T2 trabecular bone (61). While previous work has mostly focused on UTE imaging for cortical bone, this method may be used to also distinctly visualize trabecular bone, together with T2* quantification of trabecular bone (61). Hence, the role of T2* as an MRI-derived marker in osteoporosis may possibly become more important when UTE imaging using such a sequence is increasingly available.

An additive approach using quantification from CT and CSE-MRI may have higher potential for osteoporosis imaging since the two modalities provide different measures for distinct aspects of bone health, thus exploiting measures from two separate techniques as complementary information. Indeed, integrating texture analysis by adding texture features from both CT and CSE-MRI to the model with integral vBMD and PDFF increased the proportion of the explained variance for differentiating between patients with and without osteoporotic vertebral fractures from 47% to 81% according to the results of this study. Thus, when only relying on integral vBMD and PDFF as the standard measures derived from CT and CSE-MRI, a considerably larger proportion of unexplained variance may be assumed. The final model also included SRE for CT, a complex higher-order texture feature, and previous work has indicated that some GLRLM-based features that are defined over information of consecutive pixels of the same value in a given direction could be negatively associated with trabecular bone volume as measured by bone histo-morphometric evaluations (62, 63). One second-order texture feature of the final model was Variance, which is derived from GLCM and could capture image intensities and their differences of directly neighboring voxels and thereby reflect how orderly a certain microarchitecture is preserved (55). Thus, it seems reasonable that these variables were kept in the model for differentiating between patients with and without osteoporotic vertebral fractures.

To the best of our knowledge, the present study is the first to combine texture analysis for CT and CSE-MRI data to differentiate between patients with and without osteoporotic vertebral fractures. Although vertebral body segmentation as well as texture analysis are not part of the clinical routine, approaches are feasible without considerable computational efforts. In detail, CT image segmentation and vBMD extraction are already established, automated, computationally optimized, and their computational effort can therefore be considered negligible in comparison to the remaining tasks (when implementing a pipeline such as the herein used CNN-based framework for vertebral body labeling and segmentation with parameter extraction) (43–45). Details on the computational efficiency of the water-fat separation for generating PDFF and T2* maps have been reported previously for a similar workflow (49). On average, manual segmentation in PDFF maps required approximately 3 min per vertebral body, and the parallelized computation of texture features required approximately 25.5 s per vertebra for CT images and approximately 8.5 s per vertebra for PDFF maps, using a machine with 2.0 GHz CPU (Intel Core i7-8550U, 4 cores) and 16 GB RAM. However, there are limitations to this study that need to be acknowledged. First, the cohort size is small, but overall 171 vertebral bodies were included for analyses. Given our inclusion criteria that required both CT and CSE-MRI of the thoracolumbar spine within a certain period of image acquisition, eligibility was restricted to a rather small number of patients. However, we are confident that prioritizing quality and comparability of CT and MRI data over data quantity from high patient numbers is a justified approach for the purpose of this study. Second, we did not solely consider the lumbar spine (particularly L1-L3) as the site for measurements, although it is the common location for BMD assessments in clinical routine. As a consequence, we are not able to provide evidence for the common reference site for measurements exclusively. However, previous work on CT-based texture analysis has shown that improved classification of patients with and without prevalent vertebral fractures is possible by combining texture features with regional vBMD, while, importantly, all thoracolumbar levels contributed significantly to the classification (56). Third, an automatic, CNN-based approach was used for labeling and segmentation of vertebral bodies in CT images, while a manual approach was used for CSE-MRI data. According to the authors’ knowledge, accurate transfer of segmentation masks between modalities has not been achieved or made available yet, which may be related to several issues including different patient positioning during CT and MRI acquisitions, and a field of view that exhibits only a partial overlap between the two modalities in our cohort. Yet, automatic segmentation of vertebral bodies in CSE-MRI data that could be used for PDFF extraction has been described recently (64). Future work may explore applications for transfer and accurate co-registration of segmentation masks between modalities to further automatize, standardize, and accelerate medical image analyses.

## Conclusion

This study used texture analysis for CSE-MRI data of the thoracolumbar spine to predict vBMD, and to facilitate discrimination between patients with and without osteoporotic vertebral fractures. A regression model including T2* combined with three PDFF-based texture features explained 40% of the variance in integral vBMD. Further, a regression model consisting of integral vBMD and several texture features for CT and CSE-MRI data was able to predict 81% of the variance regarding the osteoporotic vertebral fracture status, compared to 47% when the model was based on integral vBMD and PDFF only. Thus, texture analysis used for advanced processing of routine CT and CSE-MRI data may improve differentiation of patients according to their fracture status when compared to vBMD and PDFF alone, which could improve prediction of the individual fracture risk in the future. Improved fracture risk prediction by using texture analysis could have important clinical implications for timely treatment initiation and prevention strategies in osteoporosis.

## Data Availability Statement

The raw data supporting the conclusions of this article will be made available by the authors, without undue reservation.

## Ethics Statement

The studies involving human participants were reviewed and approved by the Ethikkommission der Technischen Universität München. Written informed consent for participation was not required for this study in accordance with the national legislation and the institutional requirements.

## Author Contributions

Conceptualization, NS, CB, ME, SR, KS, DK, RK, TB, and MD. Methodology, NS, EAB, CB, ME, SR, EB, JK, TL, KS, DK, RK, TB, and MD. Software, NS, EAB, CB, ME, SR, and MD. Validation, NS, TB, and MD. Formal analysis, NS, EAB, CB, TB, and MD. Investigation, NS, EAB, CB, ME, SR, EB, JK, DK, TB, and MD. Resources, NS, EAB, CB, ME, SR, EB, JK, TL, KS, DK, RK, TB, and MD. Data curation, NS, CB, ME, SR, and MD. Writing—original draft preparation, NS, TB, and MD. Writing—review and editing, EAB, CB, ME, SR, EB, JK, TL, KS, DK, and RK. Visualization, NS, CB, and MD. Supervision, NS, JK, KS, DK, RK, TB, and MD. Project administration, NS, TB, and M.D. Funding acquisition, NS, JK, TL, DK, RK, TB, and MD. All authors have read and agreed to the published version of the manuscript.

## Funding

The present work was supported by the European Research Council (grant agreement No. 677661 – ProFatMRI, DK and grant agreement No. 637164 – iBack, JK), the German Research Foundation (Deutsche Forschungsgemeinschaft, DFG, project 432290010, JK and TB), the German Society of Musculoskeletal Radiology (Deutsche Gesellschaft für Muskuloskelettale Radiologie, DGMSR, NS and MD), the B. Braun Foundation (project BBST-D-19-00106, NS), and the German Academic Exchange Service (Deutscher Akademischer Austauschdienst, DAAD, NS).

## Conflict of Interest

The authors declare that the research was conducted in the absence of any commercial or financial relationships that could be construed as a potential conflict of interest.

## Publisher’s Note

All claims expressed in this article are solely those of the authors and do not necessarily represent those of their affiliated organizations, or those of the publisher, the editors and the reviewers. Any product that may be evaluated in this article, or claim that may be made by its manufacturer, is not guaranteed or endorsed by the publisher.

## Abbreviations

3D, Three-dimensional; AUC, Area under the curve; BMD, Bone mineral density; CI, Confidence interval; CNN, Convolutional neural network; CSE-MRI, Chemical shift encoding-based water-fat MRI; CT, Computed tomography; DXA, Dual-energy X-ray absorptiometry; GLCM, Gray-level co-occurrence matrix; GLRLM, Gray-level run-length matrix; HU, Hounsfield units; MRI, Magnetic resonance imaging; PACS, Picture Archiving and Communication System; PDFF, Proton density fat fraction; R²_a_, Adjusted R²; ROI, Region of interest; TE, Echo time; TR, Repetition time; UTE, Ultra-short echo-time; vBMD, Volumetric BMD.
